# Supplementary material for: Metaprofiling of the Bacterial Community in Colonized Compost Extracts by Agaricus subrufescens
Source: J Fungi (Basel). 2022 Sep 22;8(10):995. doi: 10.3390/jof8100995 (PMC9605601; doi:10.3390/jof8100995)
Supplement: Supplementary file 1 [file jof-08-00995-s001.zip › jof-1904466-supplementary.pdf]

Supplementary material

Metaprofiling of the bacterial community in colonized compost extracts by *Agaricus subrufescens*

Matheus R. Iossi[1], Isabela de Arruda Palú[2], Douglas M. M. Soares[3], Wagner Gonçalves Vieira Júnior[1], Lucas da Silva Alves[1], Cassius V. Stevani[3], Cinthia E. C. Caitano[1], Samir V. F. Atum[3,4], Renato S. Freire[3], Eustáquio S. Dias[5], Diego C. Zied[2*]

[1] Programa de Pós-Graduação em Microbiologia Agropecuária, Faculdade de Ciências Agrárias e Veterinárias (FCAV), Universidade Estadual Paulista (UNESP), Jaboticabal, São Paulo, Brazil
[2] Faculdade de Ciências Agrárias e Tecnológicas (FCAT), Universidade Estadual Paulista (UNESP), Dracena, São Paulo, Brazil
[3] Departamento de Química Fundamental, Instituto de Química, Universidade de São Paulo (USP), São Paulo, Brazil
[4] Departamento de Bioquímica, Instituto de Química, Universidade de São Paulo, São Paulo, Brazil
[5] Universidade Federal de Lavras (UFLA), Departamento de Biologia, Lavras, Minas Gerais, Brazil
*Correspondence: dczied@gmail.com or diego.zied@unesp.br (D.C.Z.)

Table S1. Mineral analysis of the compost used in the experiment.

Minerals	Concentration (g/kg)*	Organic matter (%)	pH	Moisture (%)	Carbon/Nitrogen ratio
N	22.2	71	7.2	68	18:1
P_2O_5	12.4				
K_2O	34.7				
Ca	32.9				
Mg	6.9				
S	13.0				
Na	3.7				
B	0.07				
Cu	0.22				
Fe	2.8				
Mn	0.20				
Zn	0.23				

* Mass of mineral per mass of soil.

Table S2. Abundance of bacteria present in the mushroom colonized compost extract (MCCE) obtained by immersion of the compost for 1h.

Rank	Bacteria	Reads	Rank	Bacteria	Reads
1	*Serratia marcescens*	123477	48	*Enterobacter cowanii*	382
2	*Bacillus flexus*	7461	49	*Paenibacillus alvei*	375
3	*Salmonella enterica*	5697	50	*Bacillus longiquaesitum*	370
4	*Morganella morganii*	3865	51	*Pseudomonas nitroreducens*	343
5	*Plesiomonas shigelloides*	3763	52	*Paenibacillus chondroitinus*	334
6	*Klebsiella oxytoca*	2976	53	*Micrococcus luteus*	320
7	*Paenibacillus amylolyticus*	2740	54	*Bacillus coagulans*	299
8	*Pseudomonas stutzeri*	2412	55	*Geobacillus vulcani*	287
9	*Bacillus firmus*	2294	56	*Paenibacillus stellifer*	272
10	*Trabulsiella farmeri*	2024	57	*Photorhabdus temperata*	271
11	*Paenibacillus lentimorbus*	1820	58	*Pseudomonas alcaligenes*	268
12	*Erwinia soli*	1787	59	*Serratia symbiotica*	262
13	*Anoxybacillus kestanbolensis*	1711	60	*Bacillus cohnii*	243
14	*Actinomadura vinacea*	1671	61	*Aneurinibacillus migulanus*	241
15	*Lysinibacillus boronitolerans*	1618	62	*Paenibacillus barengoltzii*	227
16	*Bacillus halodurans*	1569	63	*Staphylococcus sciuri*	222
17	*Bacillus muralis*	1480	64	*Bacillus marisflavi*	220
18	*Pseudomonas veronii*	1424	65	*Bacillus asahii*	212
19	*Bacillus thermoamylovorans*	1415	66	*Arsenophonus endosymbiont of Dermacentor variabilis*	209
20	*Bacillus humi*	1366	67	*Staphylococcus epidermidis*	198
21	*Paenibacillus mucilaginosus*	1343	68	*Thermobispora bispora*	197
22	*Nocardiopsis composta*	1317	69	*Desulfosporosinus meridiei*	180
23	*Kocuria rhizophila*	1261	70	*Pseudomonas citronellolis*	174
24	*Erwinia dispersa*	1133	71	*Staphylococcus aureus*	166
25	*Bacillus endophyticus*	1010	72	*Acinetobacter johnsonii*	158
26	*Candidatus Regiella insecticola*	974	73	*Microbacterium chocolatum*	144
27	*Pedomicrobium australicum*	965	74	*Erwinia toletana*	140
28	*Pseudomonas viridiflava*	902	75	*Corynebacterium simulans*	135
29	*Paenibacillus larvae*	898	76	*Hyphomicrobium zavarzinii*	132
30	*Bacillus oleronius*	880	77	*Faecalibacterium prausnitzii*	125
31	*Bacillus foraminis*	851	78	*Rothia dentocariosa*	121
32	*Bacillus selenatarsenatis*	785	79	*Pseudomonas balearica*	117

33	*Geobacillus thermodenitrificans*	685	80	*Rhodococcus fascians*	117
34	*Brenneria quercina*	587	81	*Virgibacillus marismortui*	114
35	*Virgisporangium ochraceum*	519	82	*Hyphomicrobium sulfonivorans*	102
36	*Ammoniphilus oxalaticus*	516	83	*Clostridium perfringens*	100
37	*Bacillus clausii*	515	84	*Sporosarcina ginsengi*	96
38	*Paenibacillus macerans*	485	85	*Brachybacterium conglomeratum*	95
39	*Propionibacterium acnes*	483	86	*Bacillus ginsengihumi*	87
40	*Sorangium cellulosum*	457	87	*Brevibacillus laterosporus*	85
41	*Paenibacillus lautus*	450	88	*Microbispora rosea*	80
42	*Corynebacterium durum*	449	89	*Bacillus safensis*	80
43	*Pseudomonas fragi*	432	90	*Nocardiopsis exhalans*	79
44	*Paenibacillus chitinolyticus*	430	91	*Pseudomonas umsongensis*	78
45	*Symbiobacterium thermophilum*	400	92	*Planococcus maitriensis*	76
46	*Brevibacillus reuszeri*	394	93	*Rothia mucilaginosa*	74
47	*Bacillus horikoshii*	392	94	*Rhodobium orientis*	72

Rank	Bacteria	Reads	Rank	Bacteria	Reads
95	*Rothia aeria*	71	145	*Arthrobacter psychrolactophilus*	13
96	*Paenibacillus illinoisensis*	66	146	*Propionibacterium granulosum*	12
97	*Bacillus farraginis*	65	147	*Providencia stuartii*	11
98	*Brevibacillus invocatus*	63	148	*Gluconacetobacter liquefaciens*	11
99	*Microbacterium maritypicum*	62	149	*Virgibacillus halodenitrificans*	11
100	*Paenibacillus curdlanolyticus*	60	150	*Corynebacterium mastitidis*	11
101	*Bacillus badius*	52	151	*Virgibacillus pantothenticus*	10
102	*Corynebacterium kroppenstedtii*	45	152	*Pseudonocardia halophobica*	10
103	*[Ruminococcus] gnavus*	43	153	*Brevundimonas diminuta*	10
104	*Nonomuraea roseoviolacea*	38	154	*Agrococcus jenensis*	10
105	*Kocuria palustris*	38	155	*Sporosarcina aquimarina*	9
106	*Oceanobacillus oncorhynchi*	37	156	*Mycobacterium llatzerense*	9
107	*Corallococcus exiguus*	37	157	*Mycobacterium arupense*	9
108	*Bacillus trypoxylicola*	37	158	*Clostridium hiranonis*	9
109	*Virgibacillus picturae*	36	159	*Staphylococcus haemolyticus*	8
110	*Desulfotomaculum aeronauticum*	36	160	*Sphingobacterium multivorum*	8
111	*Rhodoplanes elegans*	33	161	*Roseomonas mucosa*	8

112	*Veillonella dispar*	32	162	*Dorea formicigenerans*	8
113	*Microbacterium lacticum*	32	163	*Clostridium butyricum*	8
114	*Bradyrhizobium elkanii*	32	164	*Verrucosispora gifhornensis*	7
115	*Mycobacterium celatum*	31	165	*Streptomyces reticuliscabiei*	7
116	*Kurthia gibsonii*	31	166	*Ruminococcus bromii*	7
117	*Bacillus fumarioli*	31	167	*Blautia obeum*	7
118	*Paenibacillus edaphicus*	30	168	*Alkaliphilus transvaalensis*	7
119	*Brevibacterium paucivorans*	30	169	*Actinokineospora diospyrosa*	7
120	*Ruminococcus flavefaciens*	29	170	*Staphylococcus equorum*	6
121	*Rhodococcus ruber*	29	171	*Shinella granuli*	6
122	*Corynebacterium stationis*	27	172	*Salinispora tropica*	6
123	*Corynebacterium variabile*	26	173	*Saccharopolyspora hirsuta*	6
124	*Acinetobacter rhizosphaerae*	25	174	*[Ruminococcus] torques*	6
125	*Streptomyces lanatus*	25	175	*Clostridium neonatale*	6
126	*Stenotrophomonas geniculata*	22	176	*Clavibacter michiganensis*	6
127	*Acinetobacter lwoffii*	22	177	*Candidatus Aquiluna rubra*	6
128	*Brevibacillus thermoruber*	21	178	*Brevibacterium aureum*	6
129	*Acinetobacter schindleri*	20	179	*Bosea genosp.*	6
130	*Mycobacterium vaccae*	20	180	*Streptomyces radiopugnans*	5
131	*Clostridium stercorarium*	20	181	*Selenomonas noxia*	5
132	*Blautia producta*	20	182	*Nocardia concava*	5
133	*Candidatus Blochmannia floridanus*	19	183	*Citricoccus alkalitolerans*	5
134	*Veillonella parvula*	18	184	*Amycolatopsis thermoflava*	5
135	*Streptomyces mirabilis*	18	185	*Streptosporangium pseudovulgare*	4
136	*Streptomyces ahygroscopicus*	18	186	*Streptomyces scabrisporus*	4
137	*Clostridium thermopalmarium*	18	187	*Sphingobacterium mizutaii*	4
138	*Streptomyces rubrolavendulae*	17	188	*Methylosinus sporium*	4
139	*Clostridium bowmanii*	17	189	*Methylobacterium adhaesivum*	4
140	*Blastococcus aggregatus*	17	190	*Methylibium petroleiphilum*	4
141	*Nocardioides plantarum*	15	191	*Clostridium acetobutylicum*	4
142	*Janthinobacterium lividum*	15	192	*Caulobacter henricii*	4
143	*Thermoanaerobacter uzonensis*	14	193	*Bdellovibrio bacteriovorus*	4
144	*Acinetobacter guillouiae*	13	194	*Agromyces mediolanus*	4

Rank	Bacteria	Reads	Rank	Bacteria	Reads
195	*Acidovorax caeni*	4	245	*Xanthobacter autotrophicus*	1
196	*Variovorax paradoxus*	3	246	*Williamsia serinedens*	1
197	*Thermoanaerobacterium saccharolyticum*	3	247	*Streptococcus alactolyticus*	1
198	*Streptomyces aculeolatus*	3	248	*Staphylococcus succinus*	1
199	*Streptococcus anginosus*	3	249	*Staphylococcus pettenkoferi*	1
200	*Ruminococcus callidus*	3	250	*Sphingopyxis alaskensis*	1
201	*Roseateles depolymerans*	3	251	*Rhizobium leguminosarum*	1
202	*Pseudoclavibacter bifida*	3	252	*Pilimelia terevasa*	1
203	*Mycobacterium gordonae*	3	253	*Photobacterium angustum*	1
204	*Microbacterium barkeri*	3	254	*Paracoccus marcusii*	1
205	*Legionella pneumophila*	3	255	*Ochrobactrum intermedium*	1
206	*Geodermatophilus obscurus*	3	256	*Neisseria cinerea*	1
207	*Coprococcus eutactus*	3	257	*Microbacterium aurum*	1
208	*Burkholderia glathei*	3	258	*Macrococcus caseolyticus*	1
209	*Brevibacterium casei*	3	259	*Lactococcus garvieae*	1
210	*Acidovorax delafieldii*	3	260	*Lactobacillus ruminis*	1
211	*Rickettsia endosymbiont of Deronectes platynotus*	3	261	*Lactobacillus delbrueckii*	1
212	*Stenotrophomonas acidaminiphila*	2	262	*Haererehalobacter salaria*	1
213	*Sulfobacillus thermosulfidooxidans*	2	263	*Couchioplanes caeruleus*	1
214	*Streptococcus luteciae*	2	264	*Corynebacterium pilosum*	1
215	*Sporomusa polytropa*	2	265	*Clostridium tetani*	1
216	*Sphingomonas wittichii*	2	266	*Burkholderia tuberum*	1
217	*Sphingomonas azotifigens*	2	267	*Burkholderia gladioli*	1
218	*Sphingobacterium faecium*	2	268	*Bacillus acidicola*	1
219	*Rubrivivax gelatinosus*	2	269	*Asticcacaulis biprosthecium*	1
220	*Psychrobacter celer*	2	270	*Anaerospora hongkongensis*	1
221	*Paracoccus aminovorans*	2	271	*Agrobacterium sullae*	1
222	*Methylotenera mobilis*	2	272	*Aggregatibacter segnis*	1
223	*Methylobacterium organophilum*	2	273	*Actinoallomurus iriomotensis*	1
224	*Methylobacterium mesophilicum*	2	274	*Wolbachia endosymbiont of Dirofilaria immitis*	1
225	*Methylobacterium komagatae*	2	275	*Wolbachia endosymbiont of Chorthippus parallelus*	1
226	*Methylobacterium hispanicum*	2			
227	*Lactobacillus zeae*	2			
228	*Jeotgalicoccus psychrophilus*	2			

229	*Inquilinus limosus*	2
230	*Enterococcus casseliflavus*	2
231	*Cryobacterium psychrophilum*	2
232	*Corynebacterium lubricantis*	2
233	*Coprococcus catus*	2
234	*Clostridium subterminale*	2
235	*Clostridium intestinale*	2
236	*Cellulomonas xylanilytica*	2
237	*Carnobacterium viridans*	2
238	*Caldicellulosiruptor saccharolyticus*	2
239	*Burkholderia bryophila*	2
240	*Azorhizobium doebereinerae*	2
241	*Akkermansia muciniphila*	2
242	*Agrobacterium vitis*	2
243	*Acinetobacter venetianus*	1
244	*Xylanimicrobium pachnodae*	1

Table S3. Abundance of bacteria present in the mushroom colonized compost extract (MCCE) obtained by immersion of the compost for 24h.

Rank	Bacteria	Reads	Rank	Bacteria	Reads
1	*Serratia marcescens*	226018	48	*Geobacillus thermodenitrificans*	33
2	*Pseudomonas stutzeri*	22026	49	*Bacillus selenatarsenatis*	32
3	*Pseudomonas veronii*	15575	50	*Sorangium cellulosum*	31
4	*Pseudomonas viridiflava*	10383	51	*Virgisporangium ochraceum*	30
5	*Salmonella enterica*	7089	52	*Paenibacillus larvae*	29
6	*Plesiomonas shigelloides*	6252	53	*Pedomicrobium australicum*	28
7	*Pseudomonas fragi*	4769	54	*Ammoniphilus oxalaticus*	28
8	*Pseudomonas nitroreducens*	4145	55	*Symbiobacterium thermophilum*	25
9	*Morganella morganii*	3500	56	*Candidatus Blochmannia floridanus*	25
10	*Klebsiella oxytoca*	3418	57	*Bacillus clausii*	24
11	*Erwinia soli*	2967	58	*Stenotrophomonas retroflexus*	23
12	*Trabulsiella farmeri*	2853	59	*Nocardiopsis composta*	22
13	*Pseudomonas alcaligenes*	2818	60	*Paenibacillus macerans*	20

14	*Erwinia dispersa*	1249	61	*Paenibacillus chondroitinus*	20
15	*Pseudomonas balearica*	1088	62	*Bacillus horikoshii*	19
16	*Pseudomonas umsongensis*	825	63	*Bacillus coagulans*	19
17	*Brenneria quercina*	788	64	*Paenibacillus alvei*	18
18	*Candidatus Regiella insecticola*	787	65	*Staphylococcus sciuri*	18
19	*Pseudomonas citronellolis*	633	66	*Brevibacillus reuszeri*	17
20	*Acinetobacter johnsonii*	539	67	*Paenibacillus chitinolyticus*	16
21	*Enterobacter cowanii*	509	68	*Geobacillus vulcani*	16
22	*Bacillus flexus*	473	69	*Bacillus cohnii*	16
23	*Stenotrophomonas geniculata*	441	70	*Providencia stuartii*	16
24	*Photorhabdus temperata*	336	71	*Gluconacetobacter liquefaciens*	16
25	*Serratia symbiotica*	319	72	*Bacillus longiquaesitum*	14
26	*Arsenophonus endosymbiont of Dermacentor variabilis*	264	73	*Paenibacillus stellifer*	14
27	*Erwinia toletana*	172	74	*Corynebacterium durum*	13
28	*Acinetobacter rhizosphaerae*	140	75	*Bacillus marisflavi*	13
29	*Bacillus firmus*	137	76	*Micrococcus luteus*	12
30	*Paenibacillus amylolyticus*	126	77	*Aneurinibacillus migulanus*	12
31	*Bacillus muralis*	99	78	*Bacillus asahii*	12
32	*Anoxybacillus kestanbolensis*	91	79	*Thermobispora bispora*	12
33	*Lysinibacillus boronitolerans*	87	80	*Propionibacterium acnes*	11
34	*Bacillus halodurans*	85	81	*Paenibacillus lautus*	11
35	*Bacillus humi*	80	82	*Rothia dentocariosa*	10
36	*Bacillus thermoamylovorans*	79	83	*Desulfosporosinus meridiei*	8
37	*Actinomadura vinacea*	72	84	*Staphylococcus epidermidis*	7
38	*Acinetobacter lwoffii*	68	85	*Staphylococcus aureus*	7
39	*Bacillus oleronius*	67	86	*Microbacterium chocolatum*	7
40	*Paenibacillus lentimorbus*	65	87	*Corynebacterium simulans*	7
41	*Bacillus endophyticus*	63	88	*Clostridium perfringens*	7
42	*Paenibacillus mucilaginosus*	58	89	*Paenibacillus barengoltzii*	6
43	*Stenotrophomonas acidaminiphila*	52	90	*Faecalibacterium prausnitzii*	6
44	*Acinetobacter schindleri*	47	91	*Bacillus ginsengihumi*	6
45	*Bacillus foraminis*	46	92	*Rhodococcus fascians*	5
46	*Acinetobacter guillouiae*	40	93	*Virgibacillus marismortui*	5
47	*Kocuria rhizophila*	33	94	*Bacillus farraginis*	5

Rank	Bacteria	Reads	Rank	Bacteria	Reads
95	*Acinetobacter venetianus*	5	142	*Virgibacillus pantothenticus*	1
96	*Sporosarcina ginsengi*	4	143	*Mycobacterium arupense*	1
97	*Brevibacillus laterosporus*	4	144	*Clostridium hiranonis*	1
98	*Microbispora rosea*	4	145	*Staphylococcus haemolyticus*	145
99	*Bacillus safensis*	4	146	*Roseomonas mucosa*	146
100	*Rothia mucilaginosa*	4	147	*Clostridium butyricum*	147
101	*Paenibacillus curdlanolyticus*	4	148	*Ruminococcus bromii*	148
102	*Veillonella dispar*	4	149	*Alkaliphilus transvaalensis*	149
103	*Erwinia oleae*	4	150	*Streptomyces radiopugnans*	150
104	*Enterococcus sulfureus*	4	151	*Selenomonas noxia*	151
105	*Planococcus maitriensis*	3	152	*Nocardia concava*	152
106	*Rhodobium orientis*	3	153	*Streptosporangium pseudovulgare*	153
107	*Brevibacillus invocatus*	3	154	*Streptomyces scabrisporus*	154
108	*Microbacterium maritypicum*	3	155	*Clostridium acetobutylicum*	155
109	*Oceanobacillus oncorhynchi*	3	156	*Legionella pneumophila*	156
110	*Kurthia gibsonii*	3	157	*Acidovorax delafieldii*	157
111	*Bacillus fumarioli*	3	158	*Sporomusa polytropa*	158
112	*Ruminococcus flavefaciens*	3	159	*Shewanella pacifica*	159
113	*Hyphomicrobium zavarzinii*	2	160	*Selenomonas ruminantium*	160
114	*Hyphomicrobium sulfonivorans*	2	161	*Psychrobacter pulmonis*	161
115	*Rothia aeria*	2	162	*Nannocystis exedens*	162
116	*Kocuria palustris*	2	163	*Halomonas nitritophilus*	163
117	*Virgibacillus picturae*	2	164	*Haemophilus parainfluenzae*	164
118	*Desulfotomaculum aeronauticum*	2	165	*Enterococcus cecorum*	165
119	*Corynebacterium stationis*	2	166	*Allochromatium vinosum*	166
120	*Clostridium bowmanii*	2			
121	*Microbacterium barkeri*	2			
122	*Pseudoxanthomonas mexicana*	2			
123	*Clostridium subterminale*	2			
124	*Brachybacterium conglomeratum*	1			
125	*Nocardiopsis exhalans*	1			
126	*Bacillus badius*	1			
127	*Corynebacterium kroppenstedtii*	1			
128	*[Ruminococcus] gnavus*	1			

129	*Corallococcus exiguus*	1
130	*Rhodoplanes elegans*	1
131	*Brevibacterium paucivorans*	1
132	*Corynebacterium variabile*	1
133	*Brevibacillus thermoruber*	1
134	*Mycobacterium vaccae*	1
135	*Clostridium stercorarium*	1
136	*Blautia producta*	1
137	*Veillonella parvula*	1
138	*Clostridium thermopalmarium*	1
139	*Blastococcus aggregatus*	1
140	*Janthinobacterium lividum*	1
141	*Virgibacillus halodenitrificans*	1

Table S4. Agronomic traits of *A. subrufescens* mushrooms.

	Semi-controlled Conditions														
	Yield (%)			Biological Efficiency (%)			Precocity (%)			Number of Mushrooms			Weight of Mushrooms (g)		
Application date	Control	1h	24h	Control	1h	24h	Control	1h	24h	Control	1h	24h	Control	1h	24h
3rd	12.6	6.4	18.5	18.6	9.4	27.2	42.4	15.2	36.9	15	9	18	23.6	19.8	28.7
	12.9	17.3	14.4	19.0	25.4	21.2	82.3	73.1	75.4	12	21	13	30.2	23.0	31.0
	10.1	12.4	16.3	14.8	18.2	23.9	12.1	36.9	61.3	13	15	21	21.7	23.1	21.7
	18.7	12.4	19.9	27.5	18.2	29.2	56.6	51.9	38.0	19	19	22	27.5	18.3	25.3
6th	12.6	12.8	13.8	18.6	18.8	20.3	42.4	40.2	29.5	15	13	18	23.6	27.5	21.5
	12.9	19.1	19.3	19.0	28.2	28.3	82.3	56.3	64.2	12	29	23	30.2	18.5	23.4
	10.1	12.4	15.9	14.8	18.2	23.3	12.1	39.9	68.0	13	17	17	21.7	20.4	26.1
	18.7	13.1	20.4	27.5	19.3	30.0	56.6	14.1	63.8	19	15	35	27.5	24.5	16.3
9th	12.6	10.6	13.3	18.6	15.6	19.6	42.4	45.5	66.2	15	15	18	23.6	19.8	20.7
	12.9	17.7	17.3	19.0	26.0	25.5	82.3	38.0	52.6	12	21	30	30.2	23.6	16.2

	10.1	16.5	11.6	14.8	24.2	17.0	12.1	49.5	34.3	13	20	20	21.7	23.1	16.2
	18.7	16.7	17.9	18.7	24.6	26.4	56.6	63.0	34.9	19	17	27	27.5	27.5	18.6
12th	12.6	21.4	21.9	18.6	31.5	32.3	42.4	65.7	69.2	15	30	34	23.6	20.0	18.1
	12.9	13.5	21.7	19.0	19.9	31.9	82.3	61.1	57.1	12	16	31	30.2	23.6	19.6
	10.1	14.2	16.4	14.8	20.9	24.2	12.1	29.2	63.5	13	14	22	21.7	28.4	20.9
	18.7	18.8	20.1	27.5	27.7	29.6	56.6	37.2	66.5	19	27	34	27.5	19.5	16.6
15th	12.6	14.2	11.8	18.6	20.9	17.3	42.4	19.4	23.7	15	21	13	23.6	18.9	25.3
	12.9	4.4	19.7	19.0	6.5	29.0	82.3	0.0	55.1	12	8	33	30.2	15.5	16.7
	10.1	19.0	7.0	14.8	27.9	10.3	12.1	59.8	100.0	13	24	8	21.7	22.2	24.5
	18.7	16.5	18.0	27.5	24.2	26.5	56.6	68.6	52.5	19	16	23	27.5	28.8	22.0

Table S5. Mean values and standard deviation of agronomic traits of *A. subrufescens* mushrooms from the 3rd application until the 15th (see Table S2).

	Yield (%)		Precocity (%)		Number of Mushrooms		Weight of Mushrooms (g)	
	Mean	SD	Mean	SD	Mean	SD	Mean	SD
Control	14	4	48	29	15	3	15	3
1h	14	4	43	20	18	6	22	4
24h	17	4	56	18	23	8	21	4

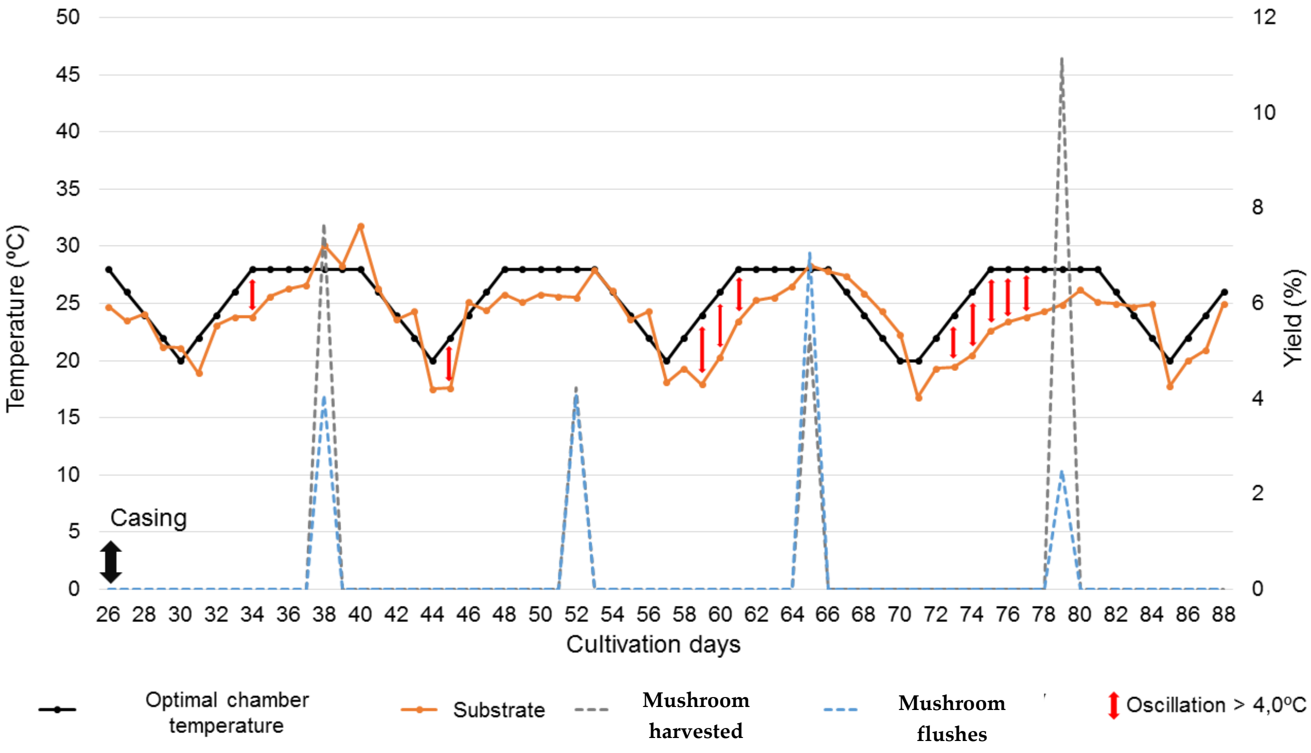

Figure S1. Chamber and compost temperature, mushroom harvested and mushroom flushes, and oscillation temperature during crop period.
